# Liver metastasis and Heng risk are prognostic factors in patients with non-nephrectomized synchronous metastatic renal cell carcinoma treated with systemic therapy

**DOI:** 10.1371/journal.pone.0211105

**Published:** 2019-02-20

**Authors:** Sung Han Kim, Jung Kwon Kim, Eun Young Park, Jungnam Joo, Kang Hyun Lee, Ho Kyung Seo, Jae Young Joung, Jinsoo Chung

**Affiliations:** 1 Department of Urology, Center for Prostate Cancer, National Cancer Center, Goyang, Korea; 2 Biometrics Research Branch, Division of Cancer Epidemiology and Prevention, Research Institute and Hospital of National Cancer Center, Goyang, Korea; University of South Alabama Mitchell Cancer Institute, UNITED STATES

## Abstract

**Objective:**

This study aimed to determine the prognostic factors of progression-free survival (PFS) and overall survival (OS) in non-nephrectomized patients with synchronous metastatic renal cell carcinoma (mRCC) receiving first-line vascular endothelial growth factor (VEGF)-targeted therapy or immunotherapy.

**Methods:**

Of 70 patients, 57 (81.4%) were treated with targeted therapy, including 5 (7.1%) with previous immunotherapy and 13 (18.6%) with immunotherapy only. The medical records of patients were retrospectively reviewed and analyzed to determine factors of PFS and OS using the Cox proportional hazards model with a statistical significance p-value <0.05.

**Results:**

The median treatment and follow-up periods were 3.9 and 30.9 months, respectively. Disease progression was reported in 90.0% of patients, with an objective response rate and clinical benefit rate of 26.1% and 76.8%, respectively. The lung (77.1%) was the most common site of metastasis. Multivariable analysis showed that poor Heng risk (hazard ratio [HR]: 2.37) and liver metastasis (HR: 2.34) were significant prognostic factors for PFS, and female sex (HR: 2.13), poor Heng risk (HR: 3.14), and liver metastasis (HR: 2.78) were significant prognostic factors for OS (p < 0.05). A subset analysis of risk factors among patients without previous history of immunotherapy also showed poor Heng risk (HR 2.92 and HR 4.24 for PFS) and liver metastasis (HR 2.87 and HR 4.81 for OS) as significant factors for both PFS and OS (p<0.05).

**Conclusion:**

Poor Heng risk, sex, and liver metastasis were associated with survival outcomes after first-line systemic therapy in patients with non-nephrectomized synchronous mRCC.

## Introduction

Global statistics suggest that approximately one-third of newly diagnosed cases of renal cell carcinoma (RCC) are detected at an advanced stage or at metastasis; this is known as synchronous metastatic renal cell carcinoma (smRCC] [1]. The prognosis of smRCC is poorer than that of metastatic recurrent RCC initially treated via radical nephrectomy; this is known as metachronous metastatic RCC (mRCC; overall survival [OS]: 4 and 19 months, respectively) [2]. The unfavorable prognosis of smRCC has been attributed to the patient’s poor general condition, which lowers tolerance for the total dose of first-line systemic therapeutic agents required. Moreover, metastatic tumors render patients ineligible for surgery, especially when critical organs are involved.

Systemic immunotherapy has been the standard therapy for mRCC over the past few decades, although with a dismal prognosis (5-year OS: <10%). With the advent of multiple molecular targeted agents, since the release of the first US Food and Drug Administration-approved agent in 2005, the standard treatment for mRCC has shifted from immunotherapy to targeted therapy as a first-line systemic therapy [3]. This change brought about an improvement in therapeutic response rates as well as longer progression-free survival (PFS) and OS durations than that observed during the immunotherapy era [4, 5].

Some immunotherapeutic agents were still in use during the targeted therapy era because of the well-documented complete response rate achieved via high-dose interleukin-2 in selected patients with mRCC and a good performance status [6]. However, thus far, the beneficial effects of targeted therapy in mRCC have been reported only for PFS and not for OS. The survival benefit from targeted therapy remains limited, with a median of <3 years despite its remarkably beneficial effects on PFS [7, 8]. Therefore, to improve the OS rate, researchers have attempted to devise the best immunotherapy and targeted therapy protocols for mRCC using accurate and significant prognostic factors.

In general, RCCs are heterotrophic solid tumors with a unique histopathology [2]. Primary renal tumors and metastatic tumors have similar but different histopathological characteristics, such that the observed response to systemic therapy is often diverse and unpredictable [1, 9]. Therefore, it is important to define the prognostic factors for mRCC in terms of OS and PFS to quickly and easily identify which patients would respond best to systemic treatment.

In this study, we aimed to determine the prognostic factors for PFS and OS using the Response Evaluation Criteria In Solid Tumors (RECIST) v1.1 in patients with naïve smRCC who did not undergo nephrectomy but received systemic first-line vascular endothelial growth factor (VEGF)-targeted therapy or immunotherapy [10, 11].

## Materials and methods

All study protocols were conducted in accordance with the ethical guidelines of the World Medical Association Declaration of Helsinki: ethical principles for medical research involving human subjects. The medical records of all enrolled patients were de-identified and analyzed anonymously. This study was approved by the Institutional Review Board of the Research Institute and Hospital National Cancer Center (NCC2016-0263). The board waived the requirement for written informed consent.

Patients with mRCC with naïve, unresectable primary renal lesions who did not undergo nephrectomy and received treatment between April 2002 and October 2015 were enrolled using the prospectively recorded RCC database of the hospital. Patients who had no eligible follow-up computed tomography (CT) imaging results during first-line systemic therapy or CT images from the last follow-up before discontinuation of treatment, discontinued systemic therapy owing to adverse side effects, refused therapy, had a past history of invasive surgical or local treatment for RCC (including nephrectomy, embolization, and radiation therapy), had bilateral RCCs, had incomplete information regarding a past history of treatment for RCC, or had a history of mTOR inhibitor-targeted treatment were excluded. The reason for excluding patients who had a history of mTOR inhibitor-targeted treatment was because the Korean National Insurance once allowed mTOR inhibitors as either first-line or second-line targeted therapy, although it was most commonly used as second-line therapy. Ultimately, 70 patients with mRCC who had not undergone nephrectomy were enrolled and followed until July 2016.

The decision to administer VEGF-targeted therapy (either sunitinib, sorafenib or pazopanib) was at the discretion of the treating urologist (J.C.) upon consideration of each patient’s histopathology, disease status, performance status, coverage by the National Health Insurance System, and the patient’s and their family’s wishes after a comprehensive discussion of the anticipated efficacy and adverse events of each agent. The targeted therapy and immunotherapy strategies and the follow-up protocols used in this study have been described previously [12]. Patients underwent a complete physical evaluation with blood tests and radiologic examinations, including CT and/or positron emission tomography-CT, as well as bone scans, to evaluate treatment response according to the RECIST (version 1.1) [11]. Treatment was continued until disease progression was detected.

The baseline characteristics and clinicopathological variables are summarized in Table 1. Continuous variables are presented as the mean and standard deviation or median (range), and categorical variables are presented as the frequency (%). PFS was defined as the period from the date of the first treatment session until progressive disease (PD), and OS was defined as the period from the date of the first treatment session to death or the last follow-up visit. Univariable and multivariable analyses were performed using the Cox proportional hazards model to investigate the potential prognostic factors for PFS and OS. Clinical variables with a p-value less than 0.2 obtained in the univariable analysis were included in the multivariable analysis, and the final model was derived using the backward selection method with an elimination criterion of p-value greater than 0.05. Furthermore, additional subgroup analyses of prognostic factors of PFS and OS among the 57 patients treated with targeted therapy only were performed. The Kaplan-Meier curve and log-rank test were used to compare the survival rate between patient groups. A two-sided p-value less than 0.05 was considered significant, and all statistical analyses were performed using SAS statistical software (version 9.3; SAS Institute Inc., Cary, NC, USA) and R-project software (version 3.3.3).

**Table 1:** Patient baseline demographics (N = 70).

Variables	n (%) unless otherwise indicated
Age (y), mean ± SD	58.77 ± 11.89
Sex	
Male	55 (78.6)
Female	15 (21.4)
Follow-up time in OS (month); median (min-max)	30.9 (6.0–30.9)
Heng risk group	
Favorable risk	1 (1.4)
Intermediate risk	43 (61.4)
Poor risk	26 (37.1)
Histology	
Clear cell type	57 (81.4)
Unclassified type	13 (18.6)
Sarcomatoid component	4 (5.7)
Anemia (Hb <13.5 for men, <12.0 for women)	49 (70.0)
Hypercalcemia	10 (14.7)
Neutrophilia	14 (20.6)
Thrombocytosis	18 (25.7)
LDH >300	14 (26.4)
KPS ≤80	6 (8.6)
WBC, median (range)	7.53 (2.95–18.07)
Albumin (g/dL), mean ± SD	3.72 ± 0.52
Clinical T stage	
T1	15 (21.4)
T2	10 (14.3)
T3	21 (30.0)
T4	6 (8.6)
Tx	18 (25.7)
Clinical N stage	
N0	24 (34.3)
N1	19 (27.1)
Nx	27 (38.6)
Number of baseline metastatic lesions	
1	25 (35.7)
2	21 (30.0)
3	18 (25.7)
4	5 (7.2)
5	1 (1.4)
Metastatic organs	
Lung	54 (77.1)
Liver	16 (22.9)
Lymph nodes	26 (37.1)
Bone	22 (31.4)
Brain	8 (11.4)
Treatment duration of previous immunotherapy (days), median (range)	67.5 (21–452)
First-line Immunotherapy	13 (18.5)
First-line Targeted agents	57 (81.4)
Sunitinib	37 (64.9)
Sorafenib	8 (14.0)
Pazopanib	12 (21.1)
Treatment duration of first line therapy (months), median (range)	3.9 (1.0–60.4)
Best overall response after first-line therapy	
PD	17 (24.3)
SD	35 (50.0)
PR	14 (20.0)
CR	4 (5.7)

SD, standard deviation; OS, overall survival; Hb, hemoglobin; LDH, lactate dehydrogenase; KPS, Karnofsky Performance Status; PD, progressive disease; PR, partial remission; CR, complete remission

## Results

Of 70 enrolled patients, 52 (74.3%) were treated with targeted therapy only and 13 (18.6%) were treated with immunotherapy only. Five (7.1%) patients who were treated with targeted therapy had history of previous immunotherapy. During a median follow-up period of 30.9 (6.0–30.9) months and a median treatment period of 3.9 (1–60.4) months, disease progression was reported in 90.0% of patients, with an objective response rate and clinical benefit rate of 26.1% and 76.8%, respectively; 5.8% of patients (n = 4) achieved complete remission. Metastasis was detected in a median of two organs with a median primary renal tumor size of 9.4 cm; the lung (77.1%) was the most common site of metastasis, followed by the lymph nodes (37.1%), bone (31.4%), liver (22.9%), and brain (12.1%). Histology results showed that 81.4% (n = 57) and 18.6% (n = 13) were clear cell type and unclassified type, respectively. A sarcomatoid component was observed in 4 (5.7%) patients. In addition, 18 (25.7%) patients had a previous history of immunotherapy, including 17 (94.4%) who were treated with interferon-alpha and 1 (5.6%) who was treated with interleukin, with a median treatment duration of 67.5 (21–452) days (Table 1).

Multivariable analysis revealed that poor Heng risk (hazard ratio [HR], 2.37; 95% confidence interval [CI], 1.37–4.10) and liver metastasis (HR 2.34, CI 1.23–4.46) were significant prognostic factors for PFS (Table 2), and female sex (HR 2.13, CI 1.13–4.05), poor Heng risk (HR 3.14, CI 1.81–5.46), and liver metastasis (HR 2.78, CI 1.42–5.41) were significant prognostic factors for OS (p < 0.05; Table 3).

**Table 2:** Cox regression analysis of the prognostic factors for progression-free survival.

	Univariable			Multivariable	
Variables	N (event)	HR (95% CI)	p-value	HR (95% CI)	p-value
Age	70 (63)	0.99 (0.96–1.01)	0.202		
Sex					
Male	55 (49)	1			
Female	15 (14)	1.35 (0.74–2.46)	0.334		
Heng risk group					
Favorable and Intermediate risk	44 (39)	1		1	
poor risk	26 (24)	2.05 (1.20–3.48)	**0.008**	2.37 (1.37–4.10)	**0.002**
WBC	70 (63)	1.06 (0.98–1.16)	0.163		
LDH	53 (48)	1.001 (1.000–1.002)	0.263		
Albumin	66 (59)	0.64 (0.40–1.02)	0.061		
Clinical stage					
T1+T2	25 (21)	1	(0.694)		
T3+T4	27 (25)	0.99 (0.55–1.77)	0.960		
Tx	17 (16)	1.28 (0.66–2.46)	0.469		
First line therapy (miss = 13)					
Sunitinib	37 (33)	1	(0.494)		
Sorafenib	8 (7)	1.60 (0.70–3.67)	0.269		
Pazopanib	12 (10)	1.28 (0.62–2.67)	0.505		
No of baseline metastatic lesions	70 (63)	1.27 (0.96–1.68)	0.101		
metastatic lesions					
Lung metastasis	54 (49)	1.55 (0.83–2.87)	0.166		
Liver metastasis	16 (14)	1.89 (1.01–3.51)	**0.045**	2.34 (1.23–4.46)	**0.010**
Lymph node metastasis	26 (23)	1.09 (0.64–1.83)	0.760		
Brain mets (miss = 4)	8 (7)	1.44 (0.65–3.21)	0.372		
Bone metastasis	22 (22)	1.20 (0.71–2.04)	0.489		

HR, hazards ratio; CI, confidence interval; LDH, lactate dehydrogenase

**Table 3:** Cox regression analysis of the prognostic factors for overall survival.

	Univariable			Multivariable	
Variables	N (event)	HR (95% CI)	p-value	HR (95% CI)	p-value
Age	70 (59)	0.99 (0.97–1.01)	0.425		
Sex					
Male	55 (45)	1		1	
Female	15 (14)	1.61 (0.88–2.95)	0.126	2.13 (1.13–4.05)	**0.020**
Heng risk group					
Favorable and Intermediate risk	44 (34)	1		1	
poor risk	26 (25)	2.63 (1.54–4.47)	**< .001**	3.14 (1.81–5.46)	**< .001**
WBC	70 (59)	1.14 (1.05–1.25)	**0.002**		
LDH	53 (45)	1.000 (0.999–1.002)	0.499		
Albumin	66 (55)	0.45 (0.27–0.73)	**0.001**		
Clinical stage					
T1+T2	25 (21)	1	(0.208)		
T3+T4	27 (23)	0.82 (0.45–1.52)	0.537		
Tx	17 (14)	1.54 (0.77–3.08)	0.218		
First line therapy					
Sunitinib	37 (31)	1	(0.186)		
Sorafenib	8 (7)	2.19 (0.95–5.09)	0.067		
Pazopanib	12 (8)	1.12 (0.51–2.47)	0.786		
No of baseline metastatic lesions	70 (59)	1.35 (1.03–1.77)	**0.031**		
metastatic lesions					
Lung	54 (45)	1.12 (0.61–2.05)	0.710		
Liver	16 (14)	1.91 (1.02–3.58)	**0.042**	2.78 (1.42–5.41)	**0.003**
Lymph node	26 (21)	1.19 (0.69–2.04)	0.527		
Brain mets	8 (7)	1.70 (0.76–3.78)	0.196		
Bone	22 (20)	1.54 (0.88–2.68)	0.129		

HR, hazards ratio; CI, confidence interval; LDH, lactate dehydrogenase; WBC, leukocytosis

Subset analyses of the prognostic factors of PFS and OS among patients with targeted agents only (with no previous immunotherapy) were performed (Tables 4 and 5). Poor Heng risk (HR 2.92, CI 1.5–5.67) and liver metastasis (HR 2.87, CI 1.35–6.12) were significant factors of PFS at multivariate analysis (p<0.05, Table 4); whereas poor Heng risk (HR 4.24, CI 2.02–8.88), leukocytosis (HR 1.18, CI 1.06–1.31), and liver metastasis (HR 4.84, CI 2.11–10.99) were significant factors of OS (p<0.05, Table 5)

**Table 4:** Cox regression analysis of the prognostic factors for progression-free survival in treated TKI only patients group.

	Univariable			Multivariable	
Variables	N (event)	HR (95% CI)	p-value	HR (95% CI)	p-value
Age	52 (46)	0.99 (0.96–1.01)	0.352		
sex					
Male	40 (35)	1			
Female	12 (11)	1.42 (0.72–2.83)	0.316		
Heng risk group					
Favorable+Intermediate risk	33 (29)	1		1	
poor risk	19 (17)	2.42 (1.27–4.61)	**0.007**	2.92 (1.5–5.67)	**0.002**
WBC	52 (46)	1.08 (0.98–1.19)	**0.118**		
LDH (miss = 13)	39 (35)	1.002 (1.000–1.004)	0.111		
Albumin (miss = 4)	48 (42)	0.67 (0.39–1.15)	0.144		
Clinical stage (miss = 1)					
T1+T2	20 (16)	1	(0.726)		
T3+T4	20 (18)	1.17 (0.59–2.33)	0.652		
Tx	11 (11)	1.37 (0.63–2.99)	0.427		
First line therapy					
Sunitinib	34 (30)	1	(0.493)		
Sorafenib	7 (6)	1.65 (0.67–4.03)	0.274		
Pazopanib	11 (10)	1.30 (0.62–2.73)	0.490		
No of baseline metastatic lesions	52 (46)	1.60 (1.14–2.25)	**0.007**		
metastatic lesions					
Lung metastasis	38 (33)	1.51 (0.78–2.94)	0.226		
Liver metastasis	13 (11)	2.21 (1.07–4.57)	**0.032**	2.87 (1.35–6.12)	**0.006**
Lymph node metastasis	20 (17)	1.22 (0.66–2.27)	0.524		
Brain mets (miss = 4)	7 (6)	1.55 (0.64–3.73)	0.332		
Bone metastasis	21 (21)	1.45 (0.80–2.62)	0.225		

HR, hazards ratio; CI, confidence interval; LDH, lactate dehydrogenase

**Table 5:** Cox regression analysis of the prognostic factors for overall survival in treated TKI only patients group.

	Univariable			Multivariable	
Variables	N (event)	HR (95% CI)	p-value	HR (95% CI)	p-value
Age	52 (41)	0.99 (0.97–1.02)	0.621		
sex					
Male	40 (30)	1			
Female	12 (11)	1.61 (0.8–3.23)	0.183		
Heng risk group					
Favorable+Intermediate risk	33 (23)	1		1	
poor risk	19 (18)	3.65 (1.90–7.03)	**< .001**	4.24 (2.02–8.88)	**< .001**
WBC	52 (41)	1.17 (1.06–1.28)	**0.001**	1.18 (1.06–1.31)	**0.003**
LDH (miss = 13)	39 (31)	1.001 (0.999–1.003)	0.477		
Albumin (miss = 4)	48 (37)	0.43 (0.24–0.75)	**0.003**		
Clinical stage (miss = 1)					
T1+T2	20 (16)	1	(0.738)		
T3+T4	20 (16)	0.95 (0.47–1.93)	0.885		
Tx	11 (8)	1.33 (0.56–3.13)	0.517		
First line therapy					
Sunitinib	34 (28)	1	(0.280)		
Sorafenib	7 (6)	2.07 (0.84–5.11)	0.114		
Pazopanib	11 (7)	1.04 (0.45–2.42)	0.920		
No of baseline metastatic lesions	52 (41)	1.51 (1.09–2.09)	**0.013**		
metastatic lesions					
Lung metastasis	38 (29)	1.26 (0.63–2.55)	0.514		
Liver metastasis	13 (11)	2.25 (1.09–4.65)	**0.029**	4.81 (2.11–10.99)	**< .001**
Lymph node metastasis	20 (15)	1.16 (0.61–2.21)	0.652		
Brain mets (miss = 4)	7 (6)	1.52 (0.63–3.67)	0.353		
Bone metastasis	21 (19)	1.70 (0.91–3.20)	0.099		

HR, hazards ratio; CI, confidence interval; LDH, lactate dehydrogenase

**Fig 1:**
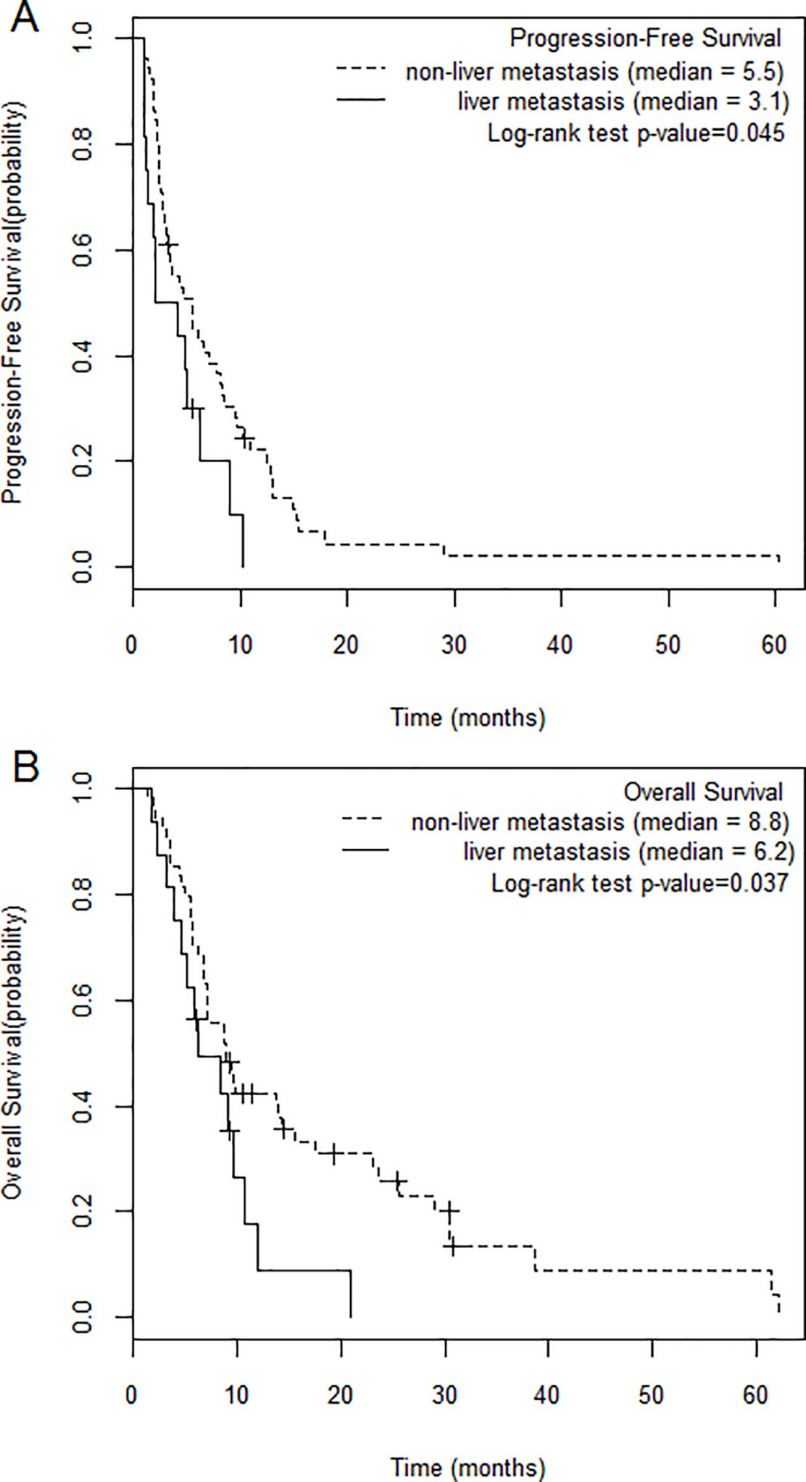
Kaplan-Meier curves of the relationship between liver metastasis and (A) progression-free survival and (B) overall survival.

Additionally, the Kaplan–Meier curve analysis showed a significant difference in PFS and OS between patients with or without liver metastasis. The median PFS duration was 3.1 (range, 1.0–10.3) months and 5.5 (range, 1.0–60.4) months in patients with or without liver metastasis, respectively. The median OS duration was 6.2 (range, 1.8–21.0) months and 8.8 (range, 1.3–62.3) months in patients with or without liver metastasis, respectively (Fig 1).

## Discussion

Considering the increase in the number of patients diagnosed with naïve unresectable synchronous mRCC, this study focused on the prognostic factors of PFS and OS during first-line TT and immunotherapy, which helps clinicians potentially identify patients who may respond best to systemic treatment quickly and easily. The present findings showed that poor Heng risk and liver metastasis were significant prognostic factors for both poor PFS and OS, and that female sex was an additional significant factor for poor OS in the multivariable analysis.

RCC with different cellular clones tends to metastasize to different organs via the bloodstream [13]. A previous study reported that hematogenous metastatic sites accounted for 80% of mRCC cases, while lymph node metastasis was reported in only 20% of cases [14]. Moreover, 20–40% of patients were likely to develop liver metastasis [14]. The prognostic significance of bone and liver metastases for survival outcomes in mRCC has been reported by the International mRCC Database Consortium (also known as the Heng risk model) study [15]: the presence of bone and liver metastasis in patients treated with targeted therapy confers a poor prognosis, and aggressive RCC subclones tend to spread to these sites. This prior study also indicated that liver metastasis was a poor prognostic factor in both PFS and OS, similar to other previous studies [15–18], whereas bone metastasis was not. In our study, liver metastasis was a significant risk factor of PFS and OS along with poor Heng risk (p<0.05, Tables 2 and 3) in mRCC patients treated with systemic therapy. The Kaplan–Meier curve analysis confirmed the significant differences in PFS and OS between patients with or without liver metastasis. Even the subgroup analyses among patients treated with targeted therapy only resulted in the same conclusion about the poorest factor of liver metastasis in mRCC (p<0.05, Tables 4 and 5). In contrast, survival outcomes have been reported to be more favorable in patients with metastasis to other organs such as the lung, pancreas, or soft tissue [19].

However, owing to the rarity of liver metastasis, a large cohort or randomized prospective study is not feasible, and subsequently, the mechanism remains unclear. A few previous studies have hypothesized that liver metastases occur in association with metastases to other sites, which is in accordance with the hematogenous spread pattern observed in RCC [20]. In fact, the incidence of solitary liver metastases in patients with mRCC has been estimated at 2–4% [20, 21]. These previous results are similar to those of our study in which the solitary bone metastasis was found only in 2 (2.8%) patients, whereas 14 other patients with bone metastases had multiple metastases (S1 Table). Consequently, the burden of hepatic tumors could represent a rate-limiting step in terms of survival outcomes.

Several studies have reported evidence of the benefits of liver-directed therapy in cases of liver metastasis in RCC (21–22). Aloia et al. [22] reported 1-, 3-, and 5-year survival rates of 46%, 24%, and 18%, respectively, in patients who received surgical treatment for liver metastasis, which is more favorable than the 2-year OS rate of 10% for patients with mRCC who do not undergo surgery. In addition, image-guided intraarterial therapies, such as transarterial chemoembolization or yttrium-90 radio-embolization, have demonstrated potential advantages for survival outcomes in these patients [1, 21]. Thus, liver-directed therapy should be considered in select mRCC patients with liver metastasis. Targeted therapy also has implications in advanced hepatocellular carcinoma. Combined treatment strategies comprising transarterial chemoembolization and sorafenib has been actively studied [23, 24] and has shown to be superior to sorafenib alone in terms of survival outcomes for hepatocellular carcinoma. However, further well-designed prospective studies are needed to establish the best multidisciplinary therapeutic protocols for mRCC with liver metastasis.

Several studies have evaluated the role of sex in survival for RCC [25–27]. Accordingly, a trend toward better survival outcomes in women has been reported. Stafford et al. hypothesized that the disparity between survival outcomes between male and female patients may derive from the biological differences in the tumor, higher prevalence of hypertension in males, and/or higher percentage of localized tumors in females [25]. However, in the mRCC, it was not always same as localized RCC. Further large cohort studies are needed to evaluate the role of sex in the survival outcomes of patients with mRCC receiving targeted therapy or immunotherapy. The current study showed that female sex was associated with unfavorable survival outcomes (Table 3). Some previous studies using animal models as well as studies of humans treated with sunitinib for mRCC showed similar prognostic outcomes as those of the current study. In a study of mice treated with sunitinib, Segarra et al. showed that male mice had a higher sunitinib concentration in the kidney, whereas female mice had a higher concentration in the liver and bone [26]. This suggests that male patients with non-nephrectomized smRCC in a primary renal tumor may have a better therapeutic response compared to that of female patients. In addition, female patients with mRCC exhibit considerably more difficulties in tolerating systemic therapies compared to those of male patients in the clinical setting. Previous studies investigating the effects of sunitinib also showed that female patients had higher rates of adverse events and lower body surface areas, which resulted in a lower tolerance for the maximal therapeutic dose, and that male patients responded better to systemic therapy for mRCC [26, 27]. Further large cohort studies are needed to evaluate the role of sex in survival of patients with mRCC treated with targeted therapy or immunotherapy in consideration for the authors’ experiences.

Poor Heng risk was found to be a significant factor for both poor PFS and OS, similar to a previous study [1, 16]. Moreover, in our previous study, the Heng risk model demonstrated marginally superior discriminatory ability than that achieved with the MSKCC model [28]. This finding strengthens the value of the Heng risk model for predicting PFS and OS in patients with naïve smRCC treated with systemic therapy. Additional subset group analysis only with patients treated with targeted therapy and without previous history of immunotherapy showed that the poor Heng risk group was significant factor for both PFS and OS (p<0.05, Tables 4 and 5).

At multivariate analysis, the differential prognostic factor for OS was found to be leukocytosis among patients treated with targeted therapy only (HR 1.13, p = 0.003; Table 5). Leukocytosis is the recognized hallmark of inflammation in cancer progression. The tumor microenvironment in which inflammatory cells are composed, partly orchestrates the oncogenetic and metastatic processes, thereby promoting tumor proliferation, survival, and migration [29]. Large numbers of granulocytes have always been observed in patients with different locally advanced and metastasized cancer. Previous study of different cancers have also confirmed that leukocytosis represents a poor risk factor of survival, similar to the finding of this study, by stimulating neutrophils to promote neoangiogenesis, suppression of systemic immunity, tumor invasion, migration, and metastasis of the tumor cells [30].

This study has a few limitations, including a small sample size, retrospective nature (although it was based on a prospectively recorded RCC database), short-term follow-up duration, and heterogeneous patient population. However, the results allow clinicians practicing at outpatient clinics to be better equipped to predict prognoses for naïve patients with unresectable smRCC. Moreover, none of the other well-known clinical factors such as T stage, age, and histopathology had a significant effect on survival outcomes, probably owing to the small population size and thereby weak statistical power. Further studies with larger sample sizes are warranted to validate these results.

## Conclusion

The findings of the present study showed that poor Heng risk, female sex, and liver metastases were associated with poor survival outcomes after first-line VEGF-targeted therapy or immunotherapy in patients with naïve, smRCC. Further well-designed prospective studies are warranted to establish the best multidisciplinary therapeutic strategy.

## Supporting information

S1 Table Concomitant incidence of multiple metastases. (DOCX)

## Abbreviations

RCC: renal cell carcinoma
PFS: progression-free survival
OS: overall survival
TT: targeted therapy
mRCC: metastatic renal cell carcinoma
PD: progressive disease
PR: partial response
SD: stable disease
HR: hazard ratio
CI: confidence interval

## References

[1] Motzer RJ. New perspectives on the treatment of metastatic renal cell carcinoma: an introduction and historical overview. Oncologist. 2011;16 Suppl 2:1–3. 10.1634/theoncologist.2011-S2-01 PMC3868198 21346034

[2] Jonasch E, Gao J, Rathmell WK. Renal cell carcinoma. BMJ. 2014;349: g4797 10.1136/bmj.g4797 25385470 PMC4707715

[3] Motzer RJ, Jonasch E, Agarwal N, Beard C, Bhayani S, Bolger GB, et al Kidney cancer, version 3.2015. J Natl Compr Canc Netw. 2015;13: 151–159. 25691606 10.6004/jnccn.2015.0022

[4] Hu B, Lara PN, Evans CP. Defining an individualized treatment strategy for metastatic renal cancer. Urol Clin North Am. 2012;39: 233–249. 10.1016/j.ucl.2012.02.002 22487765

[5] Pecuchet N, Fournier LS, Oudard S. New insights into the management of renal cell cancer. Oncology. 2013;84: 22–31. 10.1159/000342962 23076127

[6] Gills J, Parker WP, Pate S, Niu S, Van Veldhuizen P, Mirza M, et al The Role of High Dose Interleukin-2 in the Era of Targeted Therapy. J Urol. 2017;198: 538–545. 10.1016/j.juro.2017.03.076 28288839

[7] Buchler T, Bortlicek Z, Poprach A, Pavlik T, Veskrnova V, Honzirkova M, et al Outcomes for Patients with Metastatic Renal Cell Carcinoma Achieving a Complete Response on Targeted Therapy: A Registry-based Analysis. Eur Urol. 2016;70: 469–475. 10.1016/j.eururo.2015.12.031 26746623

[8] Iacovelli R, Alesini D, Palazzo A, Trenta P, Santoni M, De Marchis L, et al Targeted therapies and complete responses in first line treatment of metastatic renal cell carcinoma. A meta-analysis of published trials. Cancer Treat Rev. 2014;40: 271–275. 10.1016/j.ctrv.2013.09.003 24070900

[9] Stewart GD, Harrison DJ, Berney DM, Powles T. The molecular biology of renal cancer: another piece of the puzzle. Eur Urol. 2014;66: 85–86. 10.1016/j.eururo.2014.03.004 24674147

[10] Leon L, Garcia-Figueiras R, Suarez C, Arjonilla A, Puente J, Vargas B, et al Recommendations for the clinical and radiological evaluation of response to treatment in metastatic renal cell cancer. Target Oncol. 2014;9: 9–24. 10.1007/s11523-013-0304-7 24338498

[11] Eisenhauer EA, Therasse P, Bogaerts J, Schwartz LH, Sargent D, Ford R, et al New response evaluation criteria in solid tumours: revised RECIST guideline (version 1.1). Eur J Cancer. 2009;45: 228–247. 10.1016/j.ejca.2008.10.026 19097774

[12] Kim SH, Park WS, Joung JY, Seo HK, Lee KH, Chung J. Systemic Treatments for Metastatic Renal Cell Carcinoma: 10-Year Experience of Immunotherapy and Targeted Therapy. Cancer Res Treat. 2016;48: 1092–1101. 10.4143/crt.2015.316 26875203 PMC4946361

[13] Gerlinger M, Rowan AJ, Horswell S, Larkin J, Endesfelder D, Gronroos E, et al Intratumor heterogeneity and branched evolution revealed by multiregion sequencing. N Engl J Med. 2012;366: 883–892. 10.1056/NEJMoa1113205 22397650 PMC4878653

[14] Bianchi M, Sun M, Jeldres C, Shariat SF, Trinh QD, Briganti A, et al Distribution of metastatic sites in renal cell carcinoma: a population-based analysis. Ann Oncol. 2012;23: 973–980. 10.1093/annonc/mdr362 21890909

[15] McKay RR, Kroeger N, Xie W, Lee JL, Knox JJ, Bjarnason GA, et al Impact of bone and liver metastases on patients with renal cell carcinoma treated with targeted therapy. Eur Urol. 2014;65: 577–584. 10.1016/j.eururo.2013.08.012 23962746 PMC4123121

[16] Motzer RJ, Bukowski RM, Figlin RA, Hutson TE, Michaelson MD, Kim ST, et al Prognostic nomogram for sunitinib in patients with metastatic renal cell carcinoma. Cancer. 2008;113: 1552–8. 10.1002/cncr.23776 18720362

[17] Negrier S, Escudier B, Gomez F, Douillard JY, Ravaud A, Chevreau C, et al Prognostic factors of survival and rapid progression in 782 patients with metastatic renal carcinomas treated by cytokines: a report from the Groupe Francais d'Immunotherapie. Ann Oncol. 2002;13: 1460–1468. 12196373 10.1093/annonc/mdf257

[18] Mekhail TM, Abou-Jawde RM, Boumerhi G, Malhi S, Wood L, Elson P, et al Validation and extension of the Memorial Sloan-Kettering prognostic factors model for survival in patients with previously untreated metastatic renal cell carcinoma. J Clin Oncol. 2005;23: 832–841. 10.1200/JCO.2005.05.179 15681528

[19] Kroeger N, Choueiri TK, Lee JL, Bjarnason GA, Knox JJ, MacKenzie MJ, et al Survival outcome and treatment response of patients with late relapse from renal cell carcinoma in the era of targeted therapy. Eur Urol. 2014;65: 1086–1092. 10.1016/j.eururo.2013.07.031 23916693

[20] Alves A, Adam R, Majno P, Delvart V, Azoulay D, Castaing D, et al Hepatic resection for metastatic renal tumors: is it worthwhile? Ann Surg Oncol. 2003;10: 705–710. 12839857 10.1245/aso.2003.07.024

[21] Langan RC, Ripley RT, Davis JL, Prieto PA, Datrice N, Steinberg SM, et al Liver directed therapy for renal cell carcinoma. J Cancer. 2012;3: 184–190. 10.7150/jca.4456 22558019 PMC3342526

[22] Aloia TA, Adam R, Azoulay D, Bismuth H, Castaing D. Outcome following hepatic resection of metastatic renal tumors: the Paul Brousse Hospital experience. HPB (Oxford). 2006;8: 100–105. 18333255 10.1080/13651820500496266 PMC2131423

[23] Wang G, Liu Y, Zhou SF, Qiu P, Xu L, Wen P, et al Sorafenib combined with transarterial chemoembolization in patients with hepatocellular carcinoma: a meta-analysis and systematic review. Hepatol Int. 2016;10: 501–510. 10.1007/s12072-015-9700-7 26856326

[24] Ha Y, Lee D, Shim JH, Lim YS, Lee HC, Chung YH, et al Role of transarterial chemoembolization in relation with sorafenib for patients with advanced hepatocellular carcinoma. Oncotarget. 2016;7: 74303–74313. 10.18632/oncotarget.11030 27494871 PMC5342054

[25] Stafford HS, Saltzstein SL, Shimasaki S, Sanders C, Downs TM, Sadler GR. Racial/ethnic and gender disparities in renal cell carcinoma incidence and survival. J Urol. 2008;179: 1704–1708. 10.1016/j.juro.2008.01.027 18343443 PMC2677163

[26] Segarra I, Modamio P, Fernandez C, Marino EL. Sunitinib Possible Sex-Divergent Therapeutic Outcomes. Clin Drug Investig. 2016;36: 791–799. 10.1007/s40261-016-0428-5 27318944

[27] van der Veldt AA, Boven E, Helgason HH, van Wouwe M, Berkhof J, de Gast G, et al Predictive factors for severe toxicity of sunitinib in unselected patients with advanced renal cell cancer. Br J Cancer. 2008;99: 259–265. 10.1038/sj.bjc.6604456 18594533 PMC2480961

[28] Kwon WA, Cho IC, Yu A, Nam BH, Joung JY, Seo HK, et al Validation of the MSKCC and Heng risk criteria models for predicting survival in patients with metastatic renal cell carcinoma treated with sunitinib. Ann Surg Oncol. 2013;20: 4397–4404. 10.1245/s10434-013-3290-1 24081805

[29] Coussens LM, Werb Z. Inflammation and cancer. Nature. 2002;420: 860–867. 10.1038/nature01322 12490959 PMC2803035

[30] Dumitru CA, Lang S, Brandau S. Modulation of neutrophil granulocytes in the tumor microenvironment: Mechanisms and consequences for tumor progression. Semin Cancer Biol. 2013;23: 141–148 10.1016/j.semcancer.2013.02.005 23485549

