# Supplementary material for: Liver metastasis and Heng risk are prognostic factors in patients with non-nephrectomized synchronous metastatic renal cell carcinoma treated with systemic therapy
Source: PLoS One. 2019 Feb 20;14(2):e0211105. doi: 10.1371/journal.pone.0211105 (PMC6382149; doi:10.1371/journal.pone.0211105)
Supplement: S1 Table — (DOCX) [file pone.0211105.s001.docx]

**S1 Table.** Concomitant incidence of multiple metastases

|  | Lung | L/N | Brain | Bone | Number of patients with multiple metastasis |
| --- | --- | --- | --- | --- | --- |
| Liver (n = 16) | 12 | 7 | 3 | 3 | 14 |
| Lung (n = 54) |  | 21 | 8 | 14 | 37 |
| Lymph node (n = 26) |  |  | 4 | 9 | 25 |
| Brain (n = 8) |  |  |  | 1 | 7 |
| Bone (n = 22) |  |  |  |  | 20 |
